# Characteristics and Outcomes of Surgical Patients With Solid Cancers Admitted to the Intensive Care Unit

**DOI:** 10.1001/jamasurg.2018.1571

**Published:** 2018-06-27

**Authors:** Kathryn Puxty, Philip McLoone, Tara Quasim, Billy Sloan, John Kinsella, David S. Morrison

**Affiliations:** 1 Department of Intensive Care, Glasgow Royal Infirmary, Glasgow, United Kingdom; 2 Institute of Health and Wellbeing, University of Glasgow, Glasgow, United Kingdom; 3 School of Medicine, Glasgow Royal Infirmary, Glasgow, United Kingdom; 4 Consultant in Public Health Medicine, National Health Services Scotland, Glasgow, United Kingdom; 5 Institute of Health and Wellbeing, University of Glasgow, Glasgow, United Kingdom

## Abstract

**Importance:**

Within the surgical population admitted to intensive care units (ICUs), cancer is a common condition. However, clinicians can be reluctant to admit patients with cancer to ICUs owing to concerns about survival.

**Objective:**

To compare the clinical characteristics and outcomes of surgical patients with and without cancer who are admitted to ICUs.

**Design, Setting, and Participants:**

An observational retrospective cohort study using ICU audit records linked to hospitalization discharge summaries, cancer registrations, and death records of all 16 general adult ICUs in the West of Scotland was conducted. All 25 017 surgical ICU admissions between January 1, 2000, and December 31, 2011, were included, and data analysis was conducted during that time.

**Exposures:**

Patients were dichotomized based on a diagnosis of a solid malignant tumor as determined by its documentation in the Scottish Cancer Registry within the 2 years prior to ICU admission.

**Main Outcomes and Measures:**

Intensive care unit patients with cancer were compared with ICU patients without cancer in terms of patient characteristics (age, sex, severity of illness, reason for admission, and organ support) and survival (ICU, hospital, 6 months, and 4 years).

**Results:**

Within the 25 017 surgical ICU patients, 13 684 (54.7%) were male, the median (interquartile range [IQR]) age was 64 (50-74), and 5462 (21.8%) had an underlying solid tumor diagnosis. Patients with cancer were older (median [IQR] age, 68 [60-76] vs 62 [45-74] years; *P* < .001) with a higher proportion of elective hospitalizations (60.5% vs 19.8%; *P* < .001), similar Acute Physiology and Chronic Health Evaluation II scores (median for both, 17), but lower use of multiorgan support (57.9% vs 66.7%; *P* < .001). Intensive care unit and hospital mortality were lower for the cancer group, at 12.2% (95% CI, 11.3%-13.1%) vs 16.8% (95% CI, 16.3%-17.4%) (*P* < .001) and 22.9% (95% CI, 21.8%-24.1%) vs 28.1% (27.4%-28.7%) (*P* < .001). Patients with cancer had an adjusted odds ratio for hospital mortality of 1.09 (95% CI, 1.00-1.19). By 6 months, mortality in the cancer group was higher than that in the noncancer group at 31.3% compared with 28.2% (*P* < .001). Four years after ICU admission, mortality for those with and without cancer was 60.9% vs 39.7% (*P* < .001) respectively.

**Conclusions and Relevance:**

Cancer is a common diagnosis among surgical ICU patients and this study suggests that initial outcomes compare favorably with those of ICU patients with other conditions. Consideration that a diagnosis of cancer should not preclude admission to the ICU in patients with surgical disease is suggested.

## Key Points

**Question:**

How do features and outcomes vary for surgical patients with and without cancer admitted to the intensive care unit?

**Findings:**

In this cohort study of 25 017 surgical admissions to general intensive care units in the West of Scotland, cancer was a common morbidity at 21.8% of all admissions. Intensive care unit and hospital mortality were lower in the group of intensive care unit patients with cancer; however, this survival advantage had reversed by 6 months.

**Meaning:**

Cancer is present in 1 in 5 intensive care unit surgical admissions, and short-term survival for this group is favorable.

## Teaser

This cohort study compares admission characteristics and survival outcomes in intensive care unit patients with and without cancer who undergo surgery.

## Introduction

Up to 5% of patients with a solid malignant tumor are admitted to an intensive care unit (ICU) within 2 years of diagnosis, with most receiving organ support during their stay.^1^ Most of these patients are admitted from a surgical specialty unit, often at the time of surgical intervention for their cancer. As the incidence of cancer continues to rise,^2^ it seems likely that the number of patients with cancer who are considered for intensive care will also increase. There is some evidence that decisions to admit patients with cancer are influenced by assumptions about poor prognoses, with cancer being the second most common cause cited for ICU refusal.^3,4^ As outcomes in patients with cancer continue to improve, these assumptions may not be valid.^5^ A systematic review illustrated that variations in ICU mortality among patients with cancer were largely attributable to differences between study populations’ severity of illness, type of admission, performance status, and need for organ support rather than the presence of cancer.^6^

A limitation of many previously published studies has been that they do not include a comparison group of patients without cancer; thus, it is difficult to determine the effect of cancer within the same ICU setting.^7^ Taccone et al^8^ described the outcomes of all patients with cancer admitted to 198 European ICUs in 2002 and found that survival of patients with solid tumors was similar to that of ICU patients without cancer. More recently, a series of articles by Bos and colleagues^9,10,11^ detailed outcomes in general ICUs for patients with cancer in the Netherlands. They found that, while unplanned surgical ICU admission was associated with similar ICU mortality in patients with and without cancer, in-hospital mortality after ICU admission was higher for surgical patients with cancer than for those without cancer, at 17.4% compared with 14.6%.^10^

Considering the limited information on the comparative outcomes of patients with and without cancer admitted to general ICUs published to date, we sought to describe the characteristics and outcomes of surgical patients with solid malignant tumors following ICU admission.

## Methods

### Data Sources and Variables

This was a retrospective observational study of patients living in the West of Scotland region aged 16 years or older who were admitted to a general ICU located in the region between January 1, 2000, and December 31, 2011. Within the United Kingdom, ICU physicians have full admitting rights, although the ICU and surgical team share patient care. Full details are described elsewhere.^1^ Patients admitted from a surgical specialty at admission to the ICU were selected based on the admitting specialty recorded in the ICU database. Patients with cancer were identified as having a diagnosis of a solid malignant tumor on the Scottish Cancer Registry between January 1, 2000, and December 31, 2009. We compared these patients with cancer and surgical patients admitted to the ICU between January 1, 2000, and December 31, 2011, who did not have a preceding diagnosis of cancer on the Scottish Cancer Registry. Data analysis was conducted between January 1, 2000, and December 31, 2011, for ICU admission and January 1, 2000, and December 31, 2009, for the Scottish Cancer Registry.

This study was approved by the West of Scotland Research and Ethics Committee. Approvals to use the data were obtained from the West of Scotland Critical Care Research Network, Scottish Intensive Care Society Audit Group, and the West of Scotland Cancer Surveillance Unit. Patient identifiers were made available to the research group, but the analysis for this study was performed on an anonymized data set. Ethical review concluded that no additional patient consent would be required owing to the nature of the study.

The study used 4 linked data sets: the Scottish Cancer Registry, Scottish Morbidity Record 01, national death records, and the Scottish Intensive Care Society Audit Group WardWatcher ICU database. WardWatcher collects data on patient demographics, admitting specialty, admission diagnosis, the Acute Physiology and Chronic Health Evaluation (APACHE) II scoring system,^12^ and type of organ support. Organ support was defined as receipt of invasive mechanical ventilation, vasoactive drugs to provide cardiovascular support, or renal replacement therapy.

All surgical patients in the ICU database were included in the analysis. We used death and hospital discharge records to identify whether patients died during their hospital stay. Intensive care unit stays could not be matched to a hospital discharge summary for 649 of 25 017 patients (2.6%). For these patients, hospital discharge date and status were retrieved from the WardWatcher data set. The nature of hospital admission was unknown for these patients and they were excluded from analysis of admission type (emergency vs elective).

APACHE II scores were not recorded for 5732 patients (22.9%), and the proportion of patients with missing scores was described for both groups. For calculation of the numbers of organs supported, patients were categorized as not having received support for an organ with missing data.

### Statistical Analysis

Median and interquartile ranges (IQRs) were used to summarize continuous variables, and Wilcoxon rank sum test was applied to determine differences in median values. Pearson χ^2^ test and exact 95% CIs were used to compare proportions. Odds ratios (ORs) for hospital mortality were calculated for the presence of cancer, age 65 years or older, emergency hospitalization, direct admission from the surgical theater, reason for ICU admission, APACHE II score of 20 or higher (higher scores indicate increased severity of illness and corresponding mortality), and year of group’s ICU admission. A multivariate model was then constructed using factors with significance at *P* < .05, determined using 2-tailed, paired testing on univariate analysis with the exception of reason for ICU admission documented as *malignancy*, because this diagnosis had colinearity with the presence of cancer.

All patients were included in survival analysis. A time-varying covariate indicated the period in ICU, the stay in the hospital following discharge from the ICU, and the period following hospital discharge. Kaplan-Meier curves and log-rank test were used to compare survival between the cancer and noncancer group. Statistical analyses were performed using Stata, version 14.0 (StataCorp).

## Results

During the study period, there were 25 017 surgical patients admitted to general ICUs in the West of Scotland, of whom 13 694 (54.7%) were male. The median age was 64 years (IQR, 50-74), and 5462 (21.8%) had an underlying solid tumor diagnosis. Table 1 gives patient characteristics for surgical admissions to ICU with and without a diagnosis of cancer.

**Table 1.** Surgical Admissions to ICU in Patients With and Without Cancer^a^

Variable	All Patients			Patients Who Received Organ Support		
	Noncancer (n = 19 555)	Cancer (n = 5462)	*P* Value	Noncancer (n = 13 046)	Cancer (n = 3165)	*P* Value
Men, No. (% [95% CI])	10 696 (54.7 [54.0-55.4])	3201 (58.6 [57.3-59.9])	<.001	7312 (56.0 [55.2-56.9])	1941 (61.3 [59.6-63.0])	<.001
Median age (IQR), y	62 (45-74)	68 (60-76)	<.001	63 (46-74)	68 (60-76)	<.001
Emergency hospitalization, No./total No. (% [95% CI])	15 255/18 979 (80.2 [79.6-80.8])	2128/5389 (39.5 [38.2-40.8])	<.001	10 892/12 680 (85.9 [85.3- 86.5])	1299/3128 (41.5 [39.8-43.3])	<.001
Admitted from surgical theater, No. (% [95% CI])	12 026 (61.5 [60.8-62.2])	4375 (80.1 [79.1-81.2])	<.001	7436 (57.0 [56.2-57.9])	2329 (73.6 [72.1-75.2])	<.001
Reason for admission, No. (%)						
Malignancy	244 (1.2)	2294 (42.0)	<.001	80 (0.6)	961 (30.4)	<.001
GI/liver	4778 (24.4)	1020 (18.7)		2624 (20.1)	555 (17.5)	
Sepsis	3089 (15.8)	610 (11.2)		2949 (22.6)	540 (17.1)	
Surgical complication	893 (4.6)	376 (6.9)		689 (5.3)	297 (9.4)	
Respiratory disorder	1174 (6.0)	244 (4.5)		863 (6.6)	198 (6.3)	
Hemorrhage	1377 (7.0)	206 (3.8)		992 (7.6)	168 (5.3)	
Vascular	2392 (12.2)	56 (1.0)		1368 (10.5)	31 (1.0)	
Trauma	1702 (8.7)	30 (0.6)		1103 (8.5)	18 (0.6)	
Cardiovascular	769 (3.9)	180 (3.3)		393 (3.0)	99 (3.1)	
Renal disorder	308 (1.6)	84 (1.5)		794 (6.1)	33 (1.0)	
APACHE II score, median (IQR)	17 (12-22)	17 (13-21)	.12	18 (14-24)	18 (14-23)	.18
Not recorded, No. (%)	4073 (20.8)	1659 (30.4)	<.001	1040 (8.0)	293 (9.3)	.02
Respiratory support, No./total No. (% [95% CI])	12 300/19 220 (64.0 [63.3-64.7])	2919/5306 (55.0 [53.7-56.4])	<.001	12 300 (94.3 [93.9-94.7])	2919 (92.2 [91.2-93.1])	<.001
Unknown, No. (%)	335 (1.7)	156 (2.9)	<0.001	1	0	.62
Cardiovascular support, No./total No. (% [95% CI])	7103/19 080 (37.2 [36.4 -37.9])	1584/5291 (29.9 [28.7-31.2])	<.001	7103 (54.4 [53.7-55.4])	1584 (50.0 [48.4-51.9])	<.001
Unknown, No. (%)	475 (2.4)	171 (3.1)	.004	33 (0.3)	4 (0.1)	.18
Renal support, No./total No. (% [95% CI])	1557/16 882 (9.2 [8.8-9.7])	237/4674 (5.1 [4.5-5.7])	<.001	1557 (13.3 [12.7-14.0])	237 (8.3 [7.3-9.3])	<.001
Unknown, No. (%)	2673 (13.7)	788 (14.4)	.15	1365 (10.5)	301 (9.5)	
Organ support, No. (%)						
0	6186 (31.6)	2146 (39.3)	<.001	0	0	<.001
1	6438 (32.9)	1779 (32.6)		6438 (49.3)	1779 (56.2)	
2	5302 (27.1)	1197 (21.9)		5302 (40.6)	1197 (37.8)	
3	1306 (6.7)	189 (3.5)		1306 (10.0)	189 (6.0)	
Unknown for all modes	323 (1.7)	151 (2.8)	<.001	0	0	
ICU mortality, No. (% [95% CI])	3295 (16.8 [16.3-17.4])	666 (12.2 [11.3-13.1])	<.001	3066 (23.5 [22.8-24.2])	588 (18.6 [17.2-19.9])	<.001
Hospital mortality, No. (% [95% CI])	5490 (28.1 [27.4-28.7])	1252 (22.9 [21.8-24.1])	<.001	4693 (36.0 [35.1-36.8])	993 (31.4 [29.8-22.0])	<.001

Abbreviations: APACHE, Acute Physiology and Chronic Health Evaluation (higher scores indicate increased severity of illness and corresponding mortality); ICU, intensive care unit; IQR, interquartile range.

^a^ Numbers are cumulative total.

Intensive care unit patients with cancer tended to be older than patients without cancer with median (IQR) age 68 (60-76) vs 62 (45-74) years (*P* < .001). Most of the population without cancer had been admitted to hospital as an emergency (15 255 of 18 979 patients [80.2%]) in contrast to only 39.5% (2128 of 5389 patients) of the population with cancer. Admission to ICU directly from the surgical theater was more common in the cancer group (80.1% [4375 of 5462 patients] vs 61.5% [12 026 of 19 555 patients]; *P* < .001). Intensive care unit admission was related to an underlying solid tumor for 2294 (42.0%) of the cancer group. The most frequent diagnostic groups were otherwise similar between the cancer and noncancer groups with sepsis, gastrointestinal/ liver disease and surgical complications as common causes for admission. Vascular disease and trauma occurred more frequently in the noncancer group.

The APACHE II score was available for 15 482 (79.2%) of patients without cancer and 3803 (69.6%) of patients with cancer with similar median (IQR) values for both groups (17 [12-22] vs 17 [13-21]), *P* = .12). Organ support was provided less frequently in the cancer group compared with the noncancer group (57.9% [3165 of 5462 patients] vs 66.7% [13 046 of 19 555 patients]; *P* < .001). Single-organ support did not differ between the two groups but the provision of multi-organ support was less for the cancer group (25.4% [1386 of 5462 patients] vs 33.8% [6608 of 19 555 patients]; *P* < .001). Intensive care unit and hospital mortality were lower for the cancer population with 12.2% (666 of 5462 patients) vs 16.8% (3295 of 19 555 patients) (*P* < .001) of patients dying in ICU, and 22.9% (1252 of 5462 patients) vs 28.1% (5490 of 19 555 patients) (*P* < .001) dying in hospital.

### ICU Patients Receiving Organ Support

There were 16 211 surgical patients admitted to the ICU who received organ support during the study period (Table 1). Of these, 3165 (19.5%) had a solid tumor diagnosis. The APACHE II score was available for 92.0% of ICU patients without cancer (12 006 of 13 046) and 90.7% of ICU patients with cancer (2872 of 3165), and the median (IQR) value was 18 (14-24) and 18 (14-23). Within this group of patients, respiratory support was the most common mode of support for both the cancer and noncancer groups at 92.2% (2919 of 3165 patients) and 94.3% (12 300 of 13 046 patients), respectively. Cardiovascular support was provided to 50.0% of the cancer group (1584 of 3165 patients) and 54.6% of the noncancer group (7103 of 13 046 patients). Data pertaining to provision of renal replacement therapy was missing in 1365 patients without cancer (10.5%) and 301 patients with cancer (9.5%). Renal replacement therapy was not commonly provided in either group, but those patients in the cancer group had a lower prevalence of RRT (237 of 3165 patients [8.3%]) when compared with the noncancer group (1557 of 13 046 patients [13.3%]; *P* < .001). Single-organ support was more common in the cancer group (1779 of 3165 patients [56.2%]) with the noncancer group (6438 of 13 046 patients [49.3%]). Mortality was lower in the cancer group, with ICU mortality 18.6% (588 of 3165 patients) vs 23.5% (3066 of 13 046 patients), *P* < .001 and hospital mortality 31.4% (993 of 3165 patients) vs 36.0% (4693 of 13 065 patients), *P* < .001.

### Outcomes of Underlying Tumor Type

Table 2 lists all tumor types admitted to ICU during the study period along with ICU and hospital mortality. Short-term mortality varied considerably between different cancer types.

**Table 2.** Frequency of Tumor Types in the Surgical ICU Population and Short-term Mortality

Cancer Type	Surgical ICU Cohort, No. (%)	Mortality, % (95% CI)	
		ICU	Hospital
Colorectal	2414 (44.2)	11.6 (10.3-12.9)	21.9 (20.2-23.6)
Head and neck	610 (11.2)	5.6 (3.9-7.7)	11.0 (8.6-13.7)
Stomach	419 (7.7)	10.7 (7.9-14.1)	22.0 (18.1-26.2)
Esophagus	355 (6.5)	8.5 (5.8-11.8)	17.7 (13.9-22.1)
Kidney	230 (4.2)	9.6 (6.1-14.1)	15.2 (10.8-20.5)
Lung	220 (4.0)	35.9 (29.6-42.6)	51.4 (44.6-58.1)
Bladder	172 (3.1)	7.0 (3.7-11.9)	26.7 (20.3-34.0)
Ovary	130 (2.4)	14.6 (9.0-21.9)	29.2 (21.6-37.8)
Prostate	102 (1.9)	8.8 (4.1-16.1)	21.6 (14.0-30.8)
Uterus	102 (1.9)	10.8 (5.5-18.5)	16.7 (10.0-25.3)
Breast	99 (1.8)	15.2 (8.7-23.8)	22.2 (14.5-31.7)
Pancreas	72 (1.3)	25.0 (15.5-36.6)	47.2 (35.3-59.3)
Liver	56 (1.0)	32.1 (20.3-46.0)	58.9 (45.0-71.9)
Small intestine	50 (0.9)	14.0 (5.8-26.7)	32.0 (19.5-26.7)
Thyroid	24 (0.4)	4.2 (1.1-21.1)	8.3 (1.0-27.0)
Testis	16 (0.3)	18.8 (4.0-45.6)	18.8 (4.0-45.6)
Mesothelioma	13 (0.2)	23.1 (5.0-53.8)	46.2 (19.2-74.9)
Melanoma	11 (0.2)	0 (0-28.5)^a^	18.2 (2.3-51.8)
Other	95 (1.7)	12.6 (6.7-21.0)	25.3 (16.9-35.2)
Unknown	82 (1.5)	39.0 (28.4-50.4)	68.3 (57.1-78.1)
Multiple	190 (3.5)	8.9 (5.3-13.9)	17.4 (12.3-23.5)
Total	5462 (100)	12.2 (11.3-13.1)	22.9 (21.8-24.1)

Abbreviation: ICU, intensive care unit.

^a^ One-sided 97.5% CI.

Colorectal cancer was the commonest tumor type admitted to ICU as a surgical admission with 2414 patients (44.2%). Other common tumors included head and neck (610 patients [11.2%]) and upper gastrointestinal tract (419 patients [7.7%] with stomach cancer and 355 patients [6.5%] with esophageal cancer). Colorectal cancer had a high rate of emergency hospitalization at 45.9% (1089 of 2372 patients) with a correspondingly high median (IQR) APACHE II score of 18 (14-22) compared with that seen in head and neck tumors (median [IQR], 15 [12-19]) or esophageal cancer (median [IQR], 14 [11-19]). Organ support showed some variation by underlying tumor type. Single-organ support was common in surgical patients with head and neck cancer (467 of 610 patients [76.6%]) compared with that seen in other common tumor types (553 of 2414 patients [22.9%]) with colorectal cancer and 141 of 355 patients (39.7%) with esophageal cancer. This difference was largely accounted for by the high rate of mechanical ventilation, with 558 of 598 patients with head and neck cancer (93.3%) receiving ventilation. There was a high proportion of patients receiving no organ support in the groups with colorectal cancer (1181 of 2414 patients, 48.9%) and stomach cancer (189 of 419 patients, 45.1%). These groups also had a larger proportion of patients with missing APACHE II scores (37.6% [908 of 2414 patients] and 29.8% [125 of 419 patients], respectively).

### Factors Associated With Hospital Mortality

Hospital mortality is described for different admission features in patients with and without cancer in Table 3. Hospital mortality was lower in the cancer group when categorized by the patient’s age, severity of illness, and admission year. Mortality was higher in the cancer group for patients admitted to the hospital electively (14.8%, 95% CI, 13.6%-16.1%; vs 12.8%, 95% CI, 11.7%-13.9%; *P* = .01) and for patients admitted to the hospital as an emergency (32.7%, 95% CI, 30.7%-34.7%; vs 29.1%, 95% CI, 28.4%-29.9%; *P* = .001). Odds ratios are reported in Table 4 for factors associated with hospital mortality. The factor with the greatest association with hospital mortality was severity of illness (APACHE II score, ≥20; OR, 4.67; 95% CI, 4.34-5.01) followed by age 65 years or older (OR, 2.14; 95% CI, 2.01-2.29) and emergency hospitalization (OR, 2.86; 95% CI, 2.62-3.12). Admission to the ICU directly from the surgical theater was protective (OR, 0.53; 95% CI, 0.49-0.56). Patients with cancer had an OR of 1.09 (95% CI, 1.00-1.19) for hospital mortality after adjustment for age, hospitalization type, admission source, sepsis, APACHE II score, and year of ICU admission.

**Table 3.** Hospital Mortality in Patients With and Without Cancer by Admission Features

Variable	Patients, % (95% CI)		*P* Value
	Noncancer (n = 19 555)	Cancer (n = 5462)	
Age, y			
<65	20.0 (19.1-20.6)	15.1 (13.6-16.8)	<.001
≥65	37.8 (36.8-38.9)	27.6 (26.1-29.1)	<.001
Hospitalization			
Elective	12.8 (11.7-13.9)	14.8 (13.6-16.1)	.01
Emergency	29.1 (28.4-29.9)	32.7 (30.7-34.7)	.001
Admission from			
Surgical theater	22.3 (21.5-23.0)	17.7 (16.6-18.9)	<.001
Other	37.4 (36.3-38.5)	43.9 (40.9-46.9)	<.001
Reason for admission			
Malignancy	11.9 (8.1-16.6)	12.4 (11.1-13.8)	.81
Sepsis	40.6 (38.9-42.4)	49.0 (45.0-53.1)	<.001
Other	25.9 (25.2-26.6)	26.1 (24.4-27.9)	.85
APACHE II			
<20	15.4 (14.7-16.1)	14.1 (12.7-15.5)	.10
≥20	54.2 (52.9-55.5)	28.5 (45.8-51.3)	<.001
Unknown	23.2 (21.9-24.5)	15.6 (13.9-17.4)	<.001
ICU admission year			
2000-2003	31.9 (30.8-33.0)	23.3 (21.6-25.1)	<.001
2004-2007	27.6 (26.5-28.6)	23.5 (21.8-25.3)	<.001
2008-2011	24.4 (23.4-25.5)	20.5 (18.0-23.2)	.009

Abbreviations: APACHE, Acute Physiology and Chronic Health Evaluation (higher scores indicate increased severity of illness and corresponding mortality); ICU, intensive care unit.

**Table 4.** Multivariate Logistic Regression for Hospital Mortality

Variable	Univariate OR (95% CI)	*P* Value	Multivariate OR (95% CI)	*P* Value
Cancer	0.76 (0.71-0.82)	<.001	1.09 (1.00-1.19)	.048
Age, y				
<65	1 [Reference]		1 [Reference]	
≥65	2.28 (2.15-2.42)	<.001	2.14 (2.01-2.29)	<.001
Hospitalization				
Elective	1 [Reference]		1 [Reference]	
Emergency	2.66 (2.47-2.86)	<.001	2.86 (2.62-3.12)	<.001
Admit from				
Surgical theater	0.43 (0.41-0.46)	<.001	0.53 (0.49-0.56)	<.001
Other	1 [Reference]		1 [Reference]	
Reason for admission				
Malignancy^a^	0.40 (0.36-0.46)	<.001	NA	
Sepsis	2.06 (1.91-2.22)	<.001	1.42 (1.30-1.55)	<.001
Other	1 [Reference]		1 [Reference]	
APACHE II score				
<20	1 [Reference]		1 [Reference]	
≥20	6.35 (5.94-6.80)	<.001	4.67 (4.34-5.01)	<.001
Unknown	1.49 (1.38-1.61)	<.001	1.46 (1.34-1.59)	<.001
ICU admission year				
2000-2003	1.35 (1.25-1.45)	<.001	1.46 (1.34-1.58)	<.001
2004-2007	1.15 (1.07-1.24)	<.001	1.20 (1.10-1.31)	<.001
2008-2011	1 [Reference]		1 [Reference]	

Abbreviations: APACHE, Acute Physiology and Chronic Health Evaluation (higher scores indicate increased severity of illness and corresponding mortality); ICU, intensive care unit; NA, not applicable; OR, odds ratio.

^a^ Not included in multivariate model owing to colinearity with cancer.

### Longer-term Mortality Following ICU Admission

Longer-term survival of surgical ICU patients with and without cancer is demonstrated in the Figure. While the initial mortality associated with the critical illness appears similar, the patients in the cancer group had a higher mortality by 6 months (31.3% vs 28.2%; *P* < .001). The survival curves continue to diverge and, by 4 years, the mortality of surgical ICU patients with cancer was 60.9% compared with 39.7% seen in the group without cancer.

**Figure.**
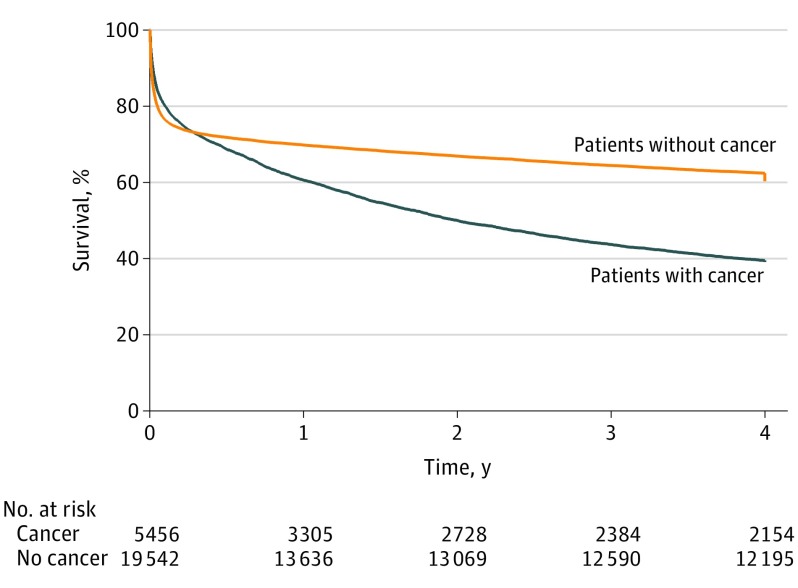
Survival Analysis of Patients With and Without Cancer Following Surgical Intensive Care Unit Admission There was a statistically significant difference in survival by log-rank test (*P* < .001).

## Discussion

In an unselected, population-based cohort, 1 in 5 surgical patients admitted to the ICU had a cancer diagnosis within 2 years of admission. These patients with cancer appeared to have an initial survival advantage over the noncancer cohort with favorable ICU and hospital mortality rates. Compared with patients without cancer, those with cancer were older and more likely to be admitted to the ICU following elective hospitalization; however, they had similar severity of illness. This finding is in keeping with those of previous studies.^8,10^

Malignancy was the commonest reason for ICU admission in the cancer group. In the noncancer cohort, 1.2% had malignancy recorded for their admission diagnosis. It is possible that this was a diagnostic error in which malignancy was suspected prior to histologic confirmation. Alternatively, patients with cancer who were not residents of Scotland may have been treated in one of the included ICUs without appearing on the Scottish Cancer Registry. Severity of illness scores for patients with and without cancer were suggestive of a similar burden of acute illness. However, multiorgan support occurred more frequently in the group of patients without cancer. This difference could be due to treatment limitations imposed on cancer patients or a lower frequency of multiorgan failure. The cancer group had a higher proportion of patients without recorded APACHE II scores, which might reflect a “well” cohort of patients admitted only for postoperative observation and therefore excluded from scoring. When the group of patients without organ support was excluded, analysis of patients admitted to the ICU for organ support revealed a similar pattern of APACHE II scores between those with and without cancer, but lower mortality in the cancer group.

In both groups, patients admitted to the hospital electively had a favorable mortality compared with those admitted to the ICU after an emergency hospitalization. This finding might be attributable to the opportunity for preoperative optimization and selection of patients without significant comorbidity for intervention. When analyzed by emergency or elective hospitalization type, mortality was higher for patients with cancer compared with the noncancer group. However, mortality for the elective admission cancer group was lower than that in the emergency admission noncancer group. We propose that the large proportion of elective hospitalizations within the cancer group has a significant association with the apparent survival advantage of patients with cancer admitted to the ICU.

After multivariate regression analysis, patients with cancer had only a marginally increased risk (OR, 1.09) of hospital mortality compared with the noncancer population. Factors that had a greater association with mortality were severity of illness, emergency hospitalization, and older age, which all increased the risk of hospital mortality, and admission directly from the surgical theater, which reduced the risk. This finding is consistent with previous studies that suggest that the immediate critical illness has a greater influence on short-term outcomes than the underlying cancer.^6^

Intensive care unit and hospital mortality varied considerably by underlying cancer type consistent with that described by Bos and colleagues.^11^ Favorable outcomes were seen for patients with thyroid, head and neck, and kidney tumors. In contrast, high ICU and hospital mortality rates were observed in patients with pancreas, lung, and liver cancer for which survival outside the ICU setting is generally poorer compared with other tumor types. Patients with an unknown tumor type had the highest mortality rates demonstrated, although this might reflect a group of patients who died prior to definitive diagnosis or those for whom further investigation would be inappropriate owing to disease burden or severe comorbidities. While clinicians should be aware that not all cancers are equal in terms of survival following surgical ICU admission, mortality rates are such that none of the tumor types should automatically preclude admission.

As more patients with cancer require critical care, clinical judgment needs to be informed by knowledge of outcomes in similar patients. The hospital mortality described for patients with cancer who are admitted to the ICU after elective hospitalization in this study is significantly higher than that described by Bos et al^9^ (14.8% vs 4.7%, respectively). However, the study by Bos et al only included patients who had a planned admission to the ICU. In comparison, patients in the present study may have had a planned admission to the hospital but required admission to the ICU only after an unexpected complication. In the same setting, surgical patients with an unplanned admission to the ICU had a hospital mortality of 17.4%,^10^ which is nearly half of that described in this study. This low mortality may be explained by the lower use of organ support in the study by Bos et al and the inclusion of patients undergoing elective surgery but admitted as an emergency after a complication. These differences highlight the importance of reporting a comparator group within the same study population to allow any real differences to be appreciated.

While immediate outcomes in this study may favor the group with cancer, this advantage was reversed by 6 months and survival thereafter was poorer in the group of patients with an underlying tumor. By 4 years, the difference in survival was 39.1% compared with 60.3% for surgical ICU patients with and without cancer. To our knowledge, no previous study has described longer-term survival for ICU patients with cancer compared with those without cancer to this degree. It has been established in the literature that short-term outcomes are related to the critical illness rather than the underlying tumor,^6^ and it seems likely that as patients recover from their critical illness, comorbidities such as cancer have an increasing association with survival in the longer term.

### Strengths and Limitations

A strength of this study is that it presents the characteristics of patients with cancer admitted to nonspecialized ICUs from a surgical population. The type of cancer was verified from cancer registration data. Our findings therefore are representative of practice in general hospitals and suitable for generalization. However, it is probable that APACHE II scores and organ support that were not recorded are not missing at random^13^ and might depend on the severity of illness, the admitting ICU, and whether the patient died during the ICU stay. Odds ratios demonstrated a slight increase in hospital mortality in patients without an APACHE II score recorded (OR, 1.46) and the reason for this is unknown. This group of patients with unrecorded APACHE II scores is likely to be a mix of those who were excluded from scoring because of a high-dependency unit admission (in which survival would be expected to be favorable) and those who died before full scoring was possible. Owing to the retrospective design of this study, we do not know the exact reason for this finding. A further limitation of this study is that our analysis was restricted to the information already collected and we were therefore unable to report on specifics, such as performance status or tumor stage, both of which are known to have a significant association with survival.

## Conclusions

We found that cancer is a common condition present in surgical patients admitted to the ICU. Patients with cancer were more likely to have been admitted to the hospital electively and receive no organ support in the ICU. Short-term outcomes in patients with cancer admitted to the ICU varied significantly by underlying tumor type, severity of illness, and admission features. Contrary to previous studies, ICU patients with cancer had favorable short-term outcomes compared with ICU patients without cancer, although this survival advantage had disappeared by 6 months. After adjusting for other prognostic variables, ICU patients with cancer did not have a meaningful increase in their risk of hospital mortality compared with patients without cancer. In view of these findings a diagnosis of cancer should not preclude admission to an ICU in surgical patients. To be able to better inform admission decisions, further work is needed on individual cancers to determine which features have prognostic value.
